# Management of pregnancy associated venous-thromboembolism: a survey of practices

**DOI:** 10.1186/1477-9560-12-12

**Published:** 2014-06-02

**Authors:** Esteban Gándara, Marc Carrier, Marc A Rodger

**Affiliations:** 1 From the Thrombosis Program, Division of Hematology, Department of Medicine, University of Ottawa-The Ottawa Hospital, Ottawa, Canada; 2 Clinical Epidemiology Unit, Ottawa Health Research Institute, Ottawa, Canada; 3 Ottawa Hospital-Ottawa Hospital Research Institute, General Campus-Centre for Practice Changing Research, 501 Smyth Road, Rm L2269e, Ottawa ONT K1H 8 L6, Canada

**Keywords:** Pregnancy, Thrombosis, Heparin

## Abstract

**Introduction:**

Low-molecular-weight heparin (LMWH) is frequently recommended for the treatment of pregnancy associated venous thromboembolism (PAVTE). Given that prior reports have suggested a wide variation in dosing of LMWH in pregnancy and the use of anti-Xa monitoring in pregnancy, the principal aim of this survey was to assess current practices for the management of PAVTE.

**Methods:**

An electronic survey was conducted. The target sample was members of the North American Society of Obstetric Medicine and Thrombosis Interest Group of Canada.

**Results:**

The final sample consisted of 27/69 hematologists (39.1%), 30/69 internists (43.5%), 8/69 obstetricians (11.6%), and 4/69 from other specialties (5.7%). For the acute treatment of patients pregnant patients with deep vein thrombosis 42/69 (60.8%) preferred LMWH given twice a day 42/69 (60.8%), whereas 25/69 (36.2%) preferred once daily. These results were similar for patients with pulmonary embolism (PE). For long-term treatment more than 70% of the respondents favoured treatment with full doses of LMWH given once a day or twice a day and 16/69 (23.2%) intermediate doses for patients diagnosed with DVT. These results were similar for patients with PE. Fourteen physicians out of 69 (20.3%) did not measure anti-Xa monitoring during acute treatment period and 24/69 (34.8%) never used anti-Xa levels during the long term treatment period. Management during the peri-partum period varied widely according to the time of the diagnosis of PAVTE.

**Discussion:**

In conclusion, our survey shows wide variation in practice regarding LMWH dosing and anti-Xa monitoring in pregnancy associated VTE and calls for trials comparing different long term strategies using LMWH in patients with PAVTE.

## Introduction

Low-molecular-weight heparin (LMWH) is frequently recommended for the treatment of pregnancy associated venous thromboembolism (PAVTE) [1]. Normal physiologic changes occurring during pregnancy affect the pharmacokinetics of LMWH that result in increasing dose requirements in pregnancy (due to a greater volume of distribution and increased renal clearance) [2]. Consequently in patients with PAVTE a more aggressive approach using continuous full doses of LMWH, given twice daily and tailored to anti-Xa levels is commonly used [3,4] during the acute treatment period (first 1–4 weeks) and long-term treatment (>1-4 weeks) without supporting evidence from high quality studies or current clinical guidelines [1]. The principal aim of this survey the principal aim of this survey was to assess current practices for the management of PAVTE.

## Methods

An electronic self-response survey, using Survey Monkey software was conducted. The target sample was experts and clinicians with experience in the management of VTE associated with pregnancy. An information sheet/electronic statement indicating that participation was voluntary and ensuring confidentiality was provided with each survey. Two groups agreed to collaborate with this project: 1-The Thrombosis Interest Group of Canada (TIGC) and 2-the International society of Obstetrics medicine (ISOM). The protocol was approved by the Ottawa Hospital Ethics and Research board to initiating the survey. The survey was pre-tested among nine thrombosis physicians at the Ottawa hospital, prior to being distributed. The evaluation of the current clinical practice was conducted using a multiple choice option format. Information regarding the type of heparin used for initial treatment and dosing; the type of heparin used for long-term-treatment and dosing; and finally the use of anti-Xa levels to guide therapy during the acute and long term treatment period was collected. Baseline characteristics of the respondents including years of practice, specialty, and knowledge about research methodology, was also captured. Finally, to ensure that the survey was answered by physicians with ample experience in the management of pregnancy associated VTE, a screening question was inserted at the beginning of the survey trying to identify physicians who have treated more than two PAVTE during the last year. This number was reached by consensus among two thrombosis experts (MR and MC).

The survey results are reported using descriptive statistics (percentages and 95% CI). Stratified analysis was conducted by years of practice, specialty, and knowledge about research methods accordingly for each question. Data was collected using Microsoft excel, and the analysis was conducted using SAS.

## Results

The survey was electronically mailed to 305 participants; 246 from the ISOM and 54 from TIGC. One hundred and five of 305 (35%) started answering the survey, of which 5 (4.5%) were disqualified after the initial screening question, and 69/305 (22.6%) completed the entire survey. The final sample consisted of 27/69 hematologists (39.1%), 30/69 internists (43.5%), 8/69 obstetricians (11.6%), and 4/69 from other specialties (5.7%). Eight out of sixty nine had practiced medicine less than 5 years, 11/69 (15.9%) between 5 to 10 years, 14/69 (20.3%) between 10 to 15 and 36/69 (52.1%) more than 15 years.

For the acute treatment of pregnant patients with deep vein thrombosis (DVT), 42/69 (60.8%) preferred LMWH given twice a day whereas 25/69 (36.2%) prescribed it once daily. These results were similar for patients with pulmonary embolism (PE), although 6% of the respondents favored UFH for the initial treatment period. More than 70% of the respondents favored treatment with full doses of LMWH given once a day or twice a day for long-term treatment (≥1 month after initiation of anticoagulation). An intermediate dose (once a day) was used by a minority physicians for long term treatment [16/69 (23.2%) for patients diagnosed with DVT and 15/69 (21.7%) for patients diagnosed with PE]. Thirty two physicians out of 69 (46.3%) reported using anti-Xa monitoring during the first 30 days in all patients and 16/69 (23.1%) only in special populations [such as extreme body weight (>150 kg or < 40 kg) or kidney disease (creatinine clearance close to 30 ml/min)] (Table 1). During long-term treatment, 47/69 (64.8%) answered that they never used anti-Xa levels or used it only in special populations, while 13/69 (18.8%) did so on a weekly or monthly basis in all patients. The use of anti-Xa monitoring varied according to the strategy used for long-term treatment. Among those using intermediate doses, 18% used some form of anti-Xa monitoring for all patients. Among those using full doses once a day 21% used some form of anti-Xa monitoring for all patients; whereas 45% of those using twice a day full doses used monitoring used some form of anti-Xa monitoring for all patients.

**Table 1:** Summary of treatment strategies, peri-partum management and anti-Xa monitoring used by respondents

		**LMWH Full dose BID**	**LMWH Full dose OD**	**UFH IV**	**UFH SC BID**	**LMWH intermediate dose OD**	**Other**
		**% (95% CI)**	**% (95% CI)**	**% (95% CI)**	**% (95% CI)**	**% (95% CI)**	**% (95% CI)**
**Acute treatment**	DVT	62.3 (50–73)	36.2 (25–48)	0	1.4 (0–7)	NA	0.14 (0–7)
	PE	62.3 (50–73)	29 (19–40)	5.8 (2–13)	1.4 (0–7)	NA	1.4 (0–7)
**Long-term treatment**	DVT	36.2 (25–48)	34.8 (24–46)	NA	0	23.2 (14–34)	5.8 (2–13)
	PE	37.7 (26–49)	36.2 (25–48)	NA	0	21.7 (13–32)	4.4 (1–11)
		**Weekly**	**Once a month**	Weekly in special populations*	Once a month in special populations*	**Never**	**Other**
		**% (95% CI)**	**% (95% CI)**	**% (95% CI)**	**% (95% CI)**	**% (95% CI)**	**% (95% CI)**
**Anti-Xa monitoring**	Acute treatment	20. 4 (12–31)	26 (16–37)	7.2 (3–15)	15.9 (9–26)	20.4 (12–31)	10.1 (4–19)
	Long-term	1.4 (0–7)	17.4 (10–27)	2.9 (0–9.2)	30.4 (20–42)	34.8 (24–46)	13.1 (24–46)
	Treatment						
		**IVC filter + LMWH BID**	**IVC filter + IV UFH**	**LMWH BID**	**UFH SC BID**	**UFH IV**	**Other**
		**% (95% CI)**	**% (95% CI)**	**% (95% CI)**	**% (95% CI)**	**% (95% CI)**	**%**
**Peri-partum Management**	VTE > 4 weeks	0	0	53.6 (41–65)	18.8 (11–29)	5.8 (2–13)	21.7 (0–9.2)
	VTE <4 and >2 weeks	0	2.9 (0–9.2)	31.9 (21–43)	10.1 (4–19)	39.1	11.7 (5–20)
	VTE <2 weeks	21.7 (15–32)	29 (19–40)	10.1 (4–19)	2.9 (0–9.2)	13.1 (6–22)	20.4 (12–31)

*Abbreviations*: *DVT* Deep vein thrombosis, *PE* Pulmonary embolism, *VTE* Venous thromboembolism, *IVC* Inferior vena cava filter, *LMWH* Low molecular weight heparin, *UFH* Unfractioned heparin, *OD* once daily, *BID* Twice daily, *IV* Intravenous, *SC* subcutaneous.

*Special populations were defined as extreme body weights >150 kg or < 40 kg or creatinine clearance close to 30 ml/min.

Management during the peri-partum period varied widely according to the time of the diagnosis of PAVTE. In those who had a diagnosis more than 4 weeks before the delivery, 37/69 preferred the use of LMWH twice daily (53.6%), whereas for those diagnosed within 2 to 4 weeks before delivery 27/69 (39.1%) physicians favored the use of IV UFH. For those diagnosed within two weeks of delivery an IVC filter in combination with LMWH or IV UFH in more was favored by 37/69 (53.2%) of the respondents.

No association was identified between physician specialty (p-value >0.05) and years of practice (p-value >0.05) with respect to dosing and the use of anti-Xa monitoring.

## Discussion

Our survey suggests that LMWH is the preferred drug for the management of PAVTE but that there is wide variation in dosing strategies and the use of anti-Xa monitoring. A wide variation in strategies used for the initial acute and long term treatment period was found for dosing (once a day vs. twice a day) and for the use of Anti-Xa monitoring corresponding to the different recommendations for anti-Xa monitoring and dosing by available guidelines guidelines [5,6] at the time that the survey was conducted. The most current recommendations by the American College of Chest Physicians (ACCP) Guidelines [1] do not support twice daily dosing or anti-Xa monitoring. One relevant finding was that nearly a quarter of the physicians used a reduced dose strategy for long-term treatment of both DVT and PE. This strategy has been recommended by experts [7] and acknowledge in the last two versions of the ACCP Guidelines [1,5] as an alternative to full dose anticoagulation in selected patients, but has never been correctly tested in this popualtion [8]. Our findings are consistent with the systematic review by Romualdi et al. [9] wide variation in the regimens used for the treatment of PAVTE. Although our findings regarding dosing strategies for long term treatment are similar to prior studies conducted by Voke et al. [3] and Knight et al. [10], our study showed decrease in the use of anti-Xa level monitoring. The study by Voke et al. reported that 76% of physicians used anti-Xa level during treatment, but did not specifically report the responses for long term treatment period. Finally most of the physicians answering the survey were not obstetricians, a phenomenon also observed in a study by Coppeltone et al. [11]. This finding may represent the current trend wherein the care of patients who develop conditions such as PAVTE during pregnancy is transferred to internal medicine specialists.

Our survey has limitations. Our response rate was low, despite multiple efforts to increase the response rate. Since the initial response rate was low (<15%) following the 2 electronic reminders, two additional measures were taken to increase the response rate: 1-the survey was endorsed by three highly respected experts; and 2-compensation in the form of a voluntary raffle. One factor that could explain our rates is that the number of studies that use e-mail to collect data has been increasing over the last years while the average response rate to the surveys appears to be decreasing [12,13]. Finally, we cannot exclude that our results reflect the practice of those with an interest in the management of PAVTE and leading to a bias interpretation of current practices.

In conclusion, our survey shows wide variation in practice regarding LMWH dosing and anti-Xa monitoring in pregnancy associated VTE and calls for trials comparing different long term strategies using LMWH in patients with PAVTE.

## Abbreviations

PAVTE: Pregnancy associated venous thromboembolism; DVT: Deep vein thrombosis; PE: Pulmonary embolism; VTE: Venous thromboembolism; IVC: Inferior vena cava filter; LMWH: Low molecular weight heparin; UFH: Unfractioned heparin; OD: Once daily; BID: Twice daily; IV: Intravenous; SC: Subcutaneous.

## Competing interests

The authors have declared that no competing interests exist.

## Authors’ contributions

Study concept and design: EG, MC and MR. Analysis and interpretation of data: EG, MC and MR. Drafting of the manuscript: EG, MC and MR. Critical revision of the manuscript for important intellectual content: EG, MC and MR. Statistical analysis: EG, MC and MR. All authors read and approved the final manuscript.
